# Designing Fe_2_O_3_-Ti as Photoanode in H-Type Double-Electrode Coupling Systems for Bidirectional Photocatalytic Production of H_2_O_2_

**DOI:** 10.3390/molecules30091908

**Published:** 2025-04-25

**Authors:** Danfeng Zhang, Changwei An, Dandan Liu, Tong Liu, Te Wang, Min Wang

**Affiliations:** 1 School of Biomedical & Chemical Engineering, Liaoning Institute of Science and Technology, Benxi 117004, China; 2 College of Chemistry, Liaoning University, Shenyang 110036, China

**Keywords:** photoelectrochemical, bidirectional coupling system, production of H_2_O_2_, Fe_2_O_3_-Ti photoanode

## Abstract

Developing high-efficiency photoelectrodes plays an important role in the photoelectrocatalytic generation of hydrogen peroxide (H_2_O_2_) in the photoelectrochemical (PEC) water splitting field. In this work, an innovative strategy was proposed, the synergistic photocatalytic production of H_2_O_2_ using a bidirectional photoanode–photocathode coupling system under visible-light irradiation. Fe_2_O_3_-Ti, as the photoanode, which was built by way of Fe_2_O_3_ loaded on Ti-mesh using the hydrothermal-calcination method, was investigated in terms of the suitability of its properties for PEC H_2_O_2_ production after optimization of the bias voltage, the type of electrolyte solution, and the concentration of the electrolyte. Afterwards, a H-type double-electrode coupling system with an Fe_2_O_3_-Ti photoanode and a WO_3_@Co_2_SnO_4_ photocathode was established for the bidirectional synergistic production of H_2_O_2_ under visible-light irradiation. The yield of H_2_O_2_ reached 919.56 μmol·L^−1^·h^−1^ in 2 h over −0.7 V with 1 mol·L^−1^ of KHCO_3_ as the anolyte and 0.1 mol·L^−1^ Na_2_SO_4_ as the catholyte (pH = 3). It was inferred that H_2_O_2_ production on the WO_3_@Co_2_SnO_4_ photocathode was in line with the 2e^-^ oxygen reduction reaction (ORR) principle, and on the Fe_2_O_3_-Ti photoanode was in line with the 2e^-^ water oxidation reaction (WOR) rule, or it was indirectly promoted by the electrolyte solution KHCO_3_. This work provides an innovative idea and a reference for anode–cathode double coupling systems for the bidirectional production of H_2_O_2_.

## 1. Introduction

Since Fujishima and Honda developed photoelectrochemical (PEC) water splitting by using a TiO_2_ photoanode in 1972, much focus has been placed on PEC as a promising technology [1]. The PEC technique for storing solar energy was deemed to be environmentally sustainable and economical, and is an efficient approach for producing hydrogen fuels by rearranging the chemical bonds of water [2,3,4,5,6]. For the PEC method, the most important challenge is to develop semiconductor photoelectrodes with high photoresponse capability and conversion capability. The photoelectrodes in PEC systems play an essential role in absorbing light to produce active charge carriers (photogenerated electron–hole pairs) for redox applications. To date, most strategies have concerned fabricating semiconductor photoelectrodes for producing and recovering H_2_ or O_2_, which has always been limited by additional bias [7,8,9]. H_2_O_2_, as the high-value-added oxidation product produced during water splitting, only generates H_2_O or O_2_ upon being utilized, and is widely applied in environmental purification, in selective organic conversion, as an energy source of fuel cells, as a cleaning agent, and so on [10,11,12]. However, little focus has been placed on the design of the photoelectrode for the PEC production of hydrogen peroxide (H_2_O_2_). The PEC technique is a high-efficiency way of preparing H_2_O_2_, mainly by way of a water oxidation reaction (WOR) and an oxygen reduction reaction (ORR) in the photoanode and photocathode. Therefore, it is valuable for creating a new type of photoelectrode to simultaneously produce H_2_O_2_ through water splitting.

In recent years, research on photoelectrodes for the production of H_2_O_2_ through PEC water splitting has been gradually increasing. BiVO_4_ photoanodes and carbon cathodes have been found to simultaneously produce electricity and H_2_O_2_ under light driving [13]. A Cu_3_BiS_3_ photocathode was used via a PEC method through indirect two-electron ORR for the visible-light-driven synthesis of H_2_O_2_ [14]. Zemin Zhang et al. [15] employed a CuBi_2_O_4_-based photocathode to achieve H_2_O_2_ production of 192.9 μmol·L^−1^. A core–shell MoSe_2_/TiO_2_ NRA photocathode was investigated for H_2_O_2_ production under ultraviolet light illumination, with a yield of 40 μmol·L^−1^·h^−1^ [16]. Two morphologically structured In_2_O_3_-coated BiVO_4_ photoanodes were effectively enhanced through selectivity and produced H_2_O_2_ via PEC water oxidation [17]. Other semiconductor materials have been used for H_2_O_2_ production through virous kinds of methods, such as noble-metal-free electrocatalysts [18], N-defect-modified g-C_3_N_4_ [19], a Na-doped BiFeO_3_ photocathode [20], a Cu^+^/Cu^2+^-substituted double perovskite Cs_2_AgBiBr_6_ photoanode [21], and so on. However, no attempts have been made to fabricate a bidirectional photocathode-and-photoanode coupling system for H_2_O_2_ evolution.

In this work, a H-type double-electrode coupling system, consisting of a WO_3_@Co_2_SnO_4_ photocathode and an Fe_2_O_3_-Ti photoanode, was designed and investigated for the bidirectional photoelectrocatalytic production of H_2_O_2_. Firstly, Fe_2_O_3_-Ti was prepared with the optimization of procedures to discuss the performance of H_2_O_2_ as the photoanode. Following this, under the best conditions, an H-type bidirectional PEC coupling system was constructed to inspect the effectiveness of the system on the synergistic production of H_2_O_2_. Simultaneously, the PEC reaction mechanism of the bidirectional coupling system was also deduced.

## 2. Discussion

### 2.1. Properties Characterization

**Figure 1:**
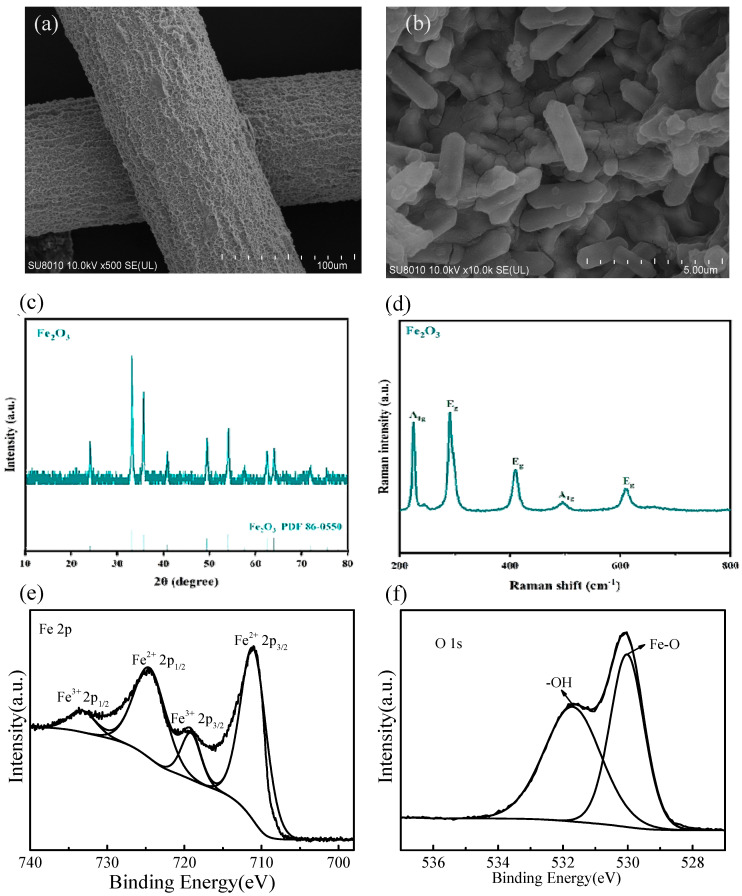
(**a**) SEM image of Ti-mesh; (**b**) SEM image of Fe_2_O_3_-Ti; (**c**) XRD of Fe_2_O_3_; (**d**) Raman spectrum of Fe_2_O_3_; XPS spectra of (**e**) Fe 2p and (**f**) O 1s of Fe_2_O_3_.

The micro morphology and structure of Ti-mesh and Fe_2_O_3_-Ti can be found in Figure 1a,b. Ti-mesh, as the photoanode substrate, displayed a network structure with a rough surface because of activation treatment. From Figure 1b, it can be seen that many short rod-like structures are attached to the base, which indicates that Fe_2_O_3_ was successfully loaded on the Ti-mesh.

The crystal structure and composition of Fe_2_O_3_ were evaluated by using XRD analysis (Figure 1c). The peaks at 2*θ* = 24.23°, 33.24°, 35.70°, 41.02°, 49.56°, 54.18°, 57.71°, 62.53°, and 64.04° correspond to the (012), (104), (110), (113), (024), (116), (018), (214), and (300) planes of Fe_2_O_3_ (JCPDS NO. 86-0550) [22], respectively. These peaks are typical characteristic diffraction peaks of α-Fe_2_O_3_. Moreover, no obvious impurity peak was detected in the XRD analysis.

The Raman spectrum of the Fe_2_O_3_ nanomaterial is displayed in Figure 1d, which is consistent with the literature [23]. The prominent peaks at 219 cm^−1^ and 495 cm^−1^ match with the A1g vibration mode, and those at 287 cm^−1^, 409 cm^−1^ and 606 cm^−1^ belong to the E_g_ vibration mode. This further proves that the Fe_2_O_3_ nanomaterial as a photoanode material was successfully prepared.

X-ray photoelectron spectroscopy (XPS) was used to further identify the chemical compositions and electronic structures of the Fe_2_O_3_ nanomaterial [24,25]. The XPS spectra of Fe-2p in Figure 1e were deconvoluted further into four peaks located at 711.03, 719.28, 724.73, and 733.28 eV, assigned to Fe^2+^ 2p _3/2_, Fe^2+^ 2p _1/2_, Fe^3+^ 2p _3/2_, and Fe^3+^ 2p _1/2_, respectively. The O 1s XPS spectrum in Figure 1f demonstrates two peaks located at 530.03 and 531.73 eV, which can be assigned to the bonds of Fe-O and -OH. The XPS results are consistent with those of the XRD and Raman analyses.

**Figure 2:**
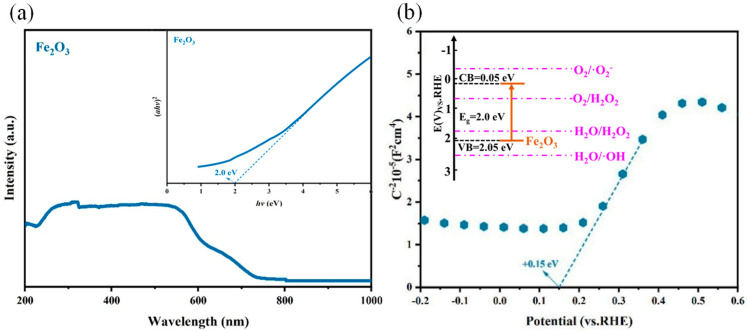
(**a**) The DRS of Fe_2_O_3_ (inset: the Kubelka-Munk plots of Fe_2_O_3_); (**b**) the Mott-Schottky plot of Fe_2_O_3_ (inset: the band gap structure of Fe_2_O_3_).

The photochemical activity of Fe_2_O_3_ was estimated using UV-Vis absorption spectroscopy, as shown in Figure 2a. The Fe_2_O_3_ nanomaterial exhibited outstanding light absorption in the wavelength range of 200–750nm. According to the calculation of the Kubelka–Munk function equation, the band gap of Fe_2_O_3_ was approximately 2.0 eV (inset of Figure 2a). The flat potential and the semiconductor type of Fe_2_O_3_ were estimated using a Mott–Schottky plot (Figure 2b). The conduction band potential (ECB) for n-type semiconductors is very close to the flat-band potential (EFB) value. In general, the value of E_FB_ for the n-type semiconductor was higher than that of E_CB_. That is, the flat-band potential was 0.1–0.3 eV higher than the ECB [26,27]. It can be estimated that Fe_2_O_3_ was an n-type semiconductor with a positive slope, and it can be confirmed that the flat-band potential (E_FB_) was 0.15 eV (vs. RHE). Thus, the E_CB_ value of Fe_2_O_3_ was +0.05 eV. The approximate band structure of Fe_2_O_3_ was as depicted in the inset of Figure 2b, from which it can be deduced that the E_VB_ of Fe_2_O_3_ met the potential (1.76 eV) for the oxidation of H_2_O to generate H_2_O_2_.

### 2.2. Photoelectrocatalytic Performance of H_2_O_2_ Production

**Figure 3:**
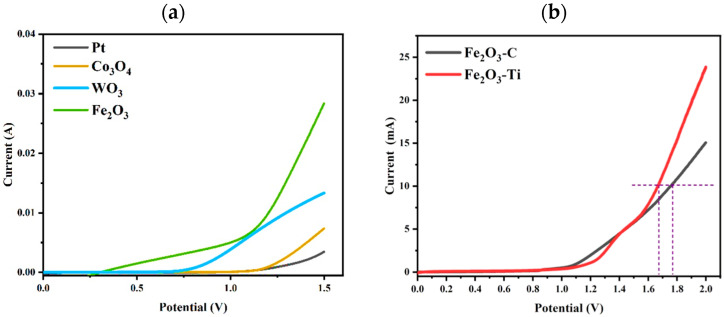
(**a**) LSV spectra of different photoanodes; (**b**) LSV spectra of Fe_2_O_3_-C and Fe_2_O_3_-Ti (reaction conditions: 1 mol·L^−1^ of KHCO_3_ (20 mL), 20 °C, 2V (vs. Ag/AgCl), 300 W Xe (λ ≥ 420 nm) illumination).

When the anode was used for WOR, the formation of O_2_ via 4e^-^ WOR competed with the production of H_2_O_2_ via 2e^-^ WOR. The potential for water oxidation to produce H_2_O_2_ was higher than that of O_2_, which made it more difficult to achieve the selective generation of H_2_O_2_. Therefore, it is important to choose one appropriate semiconductor material as the anode so that E_g_ can meet the potential of 2e^-^ WOR. Because of these reasons, the linear scanning voltammetry (LSV) curves of different anode materials (Pt, WO_3_, Co_3_O_4_, and Fe_2_O_3_) were investigated, and the current changes at different potentials were explored in detail. As shown in Figure 3a, with an increase in voltage, the current via different anodes gradually increased; that is, Fe_2_O_3_ > WO_3_ > Co_3_O_4_ > Pt. From Figure 3a, Fe_2_O_3_, when used as the anode material, displayed the lowest onset potential and highest current density. This was the reason for choosing Fe_2_O_3_ as the photoanode material. Figure 3b shows the LSV curves of Fe_2_O_3_-C (Fe_2_O_3_ dripped onto carbon paper) and Fe_2_O_3_-Ti as the electrode. While the onset potential of Fe_2_O_3_-Ti, so far, was higher than that of Fe_2_O_3_-C, Fe_2_O_3_-Ti only needed 1.68 V, which was lower than the onset potential of Fe_2_O_3_-C (1.78V) when the current reached 10 mA. In other words, Fe_2_O_3_-Ti, when used as the electrode, required much lower power consumption, which meant it was more suitable for Fe_2_O_3_-Ti when used as the photoanode material in the whole application.

**Figure 4:**
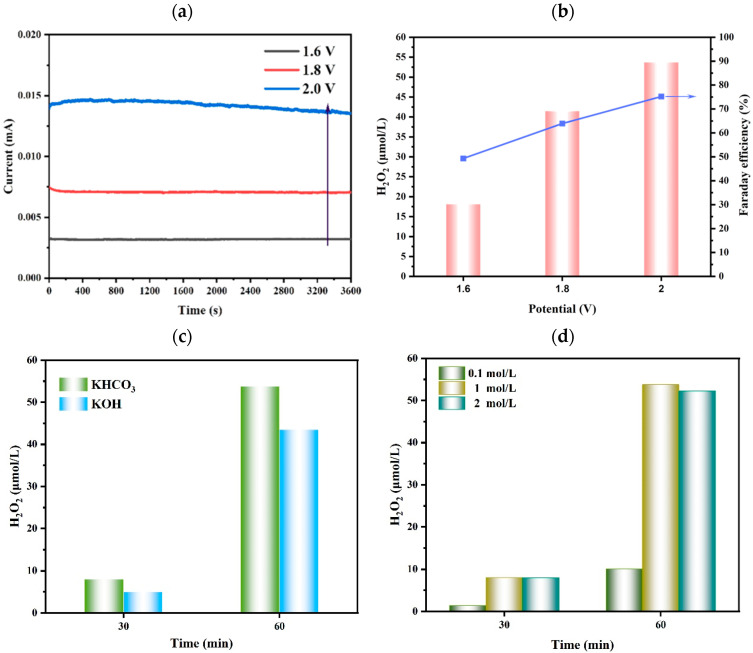
Optimization of (**a**) photocurrents at different potentials of Fe_2_O_3_-Ti photoanode, (**b**) Faraday efficiency at different potentials of Fe_2_O_3_-Ti photoanode, (**c**) different types of electrolyte solution, and (**d**) concentrations of electrolyte solution (reaction conditions: 1 mol·L^−1^ of KHCO_3_ (20 mL), 20 °C, 2V (vs. Ag/AgCl), 300 W Xe (λ ≥ 420 nm) illumination).

The anodic potentials, the type of electrolyte solution, and the concentration of electrolyte solution were all optimized, as shown in Figure 4. The photocurrents of the Fe_2_O_3_-Ti photoanode at different potentials are shown in Figure 4a; the photocurrent at 2.0V was 2–3 times higher than that at 1.6 V and 1.8 V. Meanwhile, the production of H_2_O_2_ with the Faraday efficiency at nearly 75.2%, at 2.0 V, was higher than that of the others (Figure 4b). Figure 4c shows that the yield of H_2_O_2_ using KHCO_3_ as the electrolyte solution obviously surpassed that when using KOH as the electrolyte solution. And when the concentration of KHCO_3_ was 1 mol·L^−1^, the yield of H_2_O_2_ was higher compared to the other concentrations (0.1 mol·L^−1^ and 2 mol·L^−1^). Therefore, the optimal conditions for the production of H_2_O_2_ were 2.0 V as the anode potential and 1 mol·L^−1^ of KHCO_3_ as the electrolyte solution, which applied to the whole photoelectrocatalysis process.

**Figure 5:**
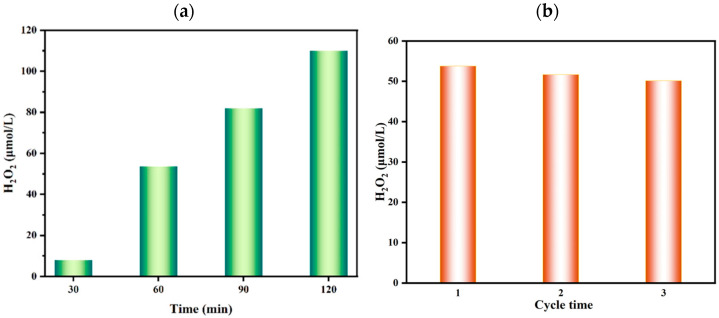
(**a**) Yield of H_2_O_2_ at different times on Fe_2_O_3_-Ti photoanode under optimal conditions; (**b**) cycle stability of Fe_2_O_3_-Ti photoanode (reaction conditions: 1 mol·L^−1^ of KHCO_3_ (20 mL), 20 °C, 2V (vs. Ag/AgCl), 300 W Xe (λ ≥ 420 nm) illumination).

Based on the above optimal conditions, the continuous production of H_2_O_2_ using the photoanode were investigated. It is clearly presented in Figure 5a that the cumulative concentration of H_2_O_2_ produced by the Fe_2_O_3_-Ti photoanode within two hours using 1 mol·L^−1^ of KHCO_3_ as the electrolyte solution was 110.27 μmol·L^−1^. Figure 5b demonstrates that the cyclic stabilization of the Fe_2_O_3_-Ti photoanode was performed for 1h three times. After the third time, the yield of H_2_O_2_ only decreased by 6.8%, indicating the outstanding stability of the Fe_2_O_3_-Ti electrode as the photoanode material.

### 2.3. Synergistic Photocatalytic Production of H_2_O_2_ by Bidirectional Coupling System

On account of the satisfactory performance of WO_3_@Co_2_SnO_4_ as the photocathode (which was discussed in the previous paper [22]) and Fe_2_O_3_-Ti as the photoanode, a bidirectional coupling system was established for the synergistic photocatalytic production of H_2_O_2_ within a H-type quartz cell. The cell structure was as follows: a WO_3_@Co_2_SnO_4_ photocathode at one side of the H-type quartz cell with 0.1 mol·L^−1^ Na_2_SO_4_ as the catholyte (pH = 3), an Fe_2_O_3_-Ti photoanode on the other side with 1 mol·L^−1^ of KHCO_3_ as the anolyte, and a Ag/AgCl electrode as the reference electrode on the side of the photocathode. A proton exchange membrane (PEM) was used to divide the anode and cathode. Before the photoelectrocatalytic reaction, O_2_ gas was purged on both sides of the H-type reactor to ensure adsorption–desorption equilibrium.

**Figure 6:**
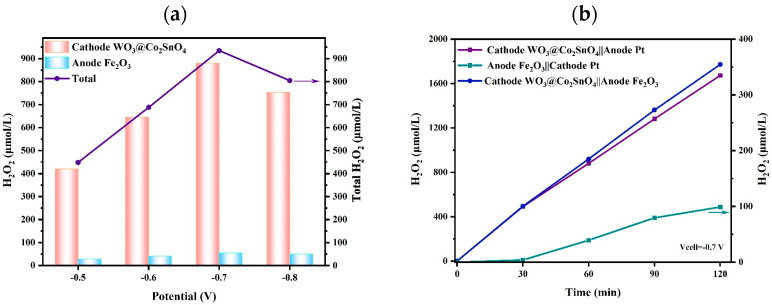
(**a**) The yield of H_2_O_2_ at different potentials; (**b**) the yield of H_2_O_2_ on the bidirectional coupling system over time (reaction conditions: a H-type quartz reactor with 300 W Xe light (λ ≥ 420 nm) as the visible-light source, a bias voltage of -0.7 V (vs. Ag/AgCl), 20 mL of KHCO_3_ (1 mol·L^−1^), and 20 mL of Na_2_SO_4_ (0.1 mol·L^−1^, pH = 3) were used as the electrolyte solution for the anode and cathode).

As given in Figure 6a, different potentials, that is, −0.5 V, −0.6 V, −0.7 V, and −0.8 V, were inspected. When the applied voltage across the cell electrodes was at −0.7 V, the yield of H_2_O_2_ was the highest for both the WO_3_@Co_2_SnO_4_ photocathode and the Fe_2_O_3_-Ti photoanode. Then, under a potential of −0.7 V, the synergistic photocatalytic production of H_2_O_2_ using a bidirectional cathode–anode coupling system was carried out for 2 h, as shown in Figure 6b, which shows that the concentration of H_2_O_2_ reached 1672.7 μmol·L^−1^ at the WO_3_@Co_2_SnO_4_ photocathode and 98.84 μmol·L^−1^ at the Fe_2_O_3_-Ti photoanode. It is obvious that the Fe_2_O_3_-Ti photoanode and the WO_3_@Co_2_SnO_4_ photocathode can be applied as an effective electrode couple for the production of H_2_O_2_.

**Scheme 1:**
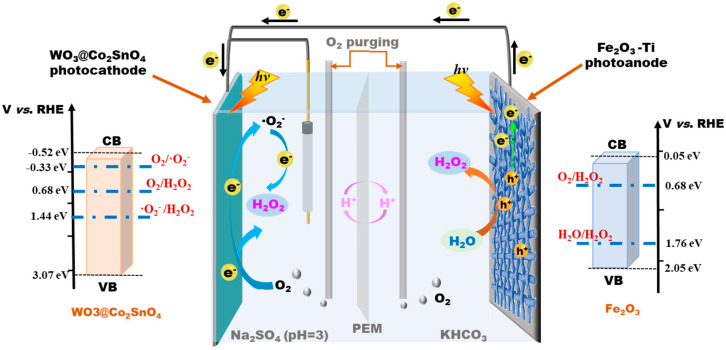
The deduced mechanism of the bidirectional PEC production of H_2_O_2_ under the double photoanode-photocathode coupling system.

According to the above results, the Synergistic photocatalytic reaction mechanism of the bidirectional coupling system was deduced. As demonstrated in Scheme 1, for the WO_3_@Co_2_SnO_4_ photocathode, the production of H_2_O_2_ followed the 2e^-^ ORR process. The conduction band (CB) edge of WO_3_@Co_2_SnO_4_ was around −0.52 eV [22], which was more negative than the potential of O_2_/O_2_^-^ (−0.33 eV). And its valance band (VB) edge was more positive than the potential of O_2_/H_2_O_2_ (0.68 eV) and O_2_^-^/H_2_O_2_ (1.44 eV). This means the WO_3_@Co_2_SnO_4_ photocathode absorbed the incident light to form photogenerated electron-hole pairs (e^-^-h^+^). The photogenerated e^-^ reacted with O_2_ to generate H_2_O_2_ (R3) and also create a superoxide radical (O_2_^-^); then, O_2_^-^ continued to produce H_2_O_2_ (R4). On the other hand, H_2_O_2_ production on the Fe_2_O_3_-Ti photoanode was inferred to follow the rule of 2e^-^ WOR, or was indirectly promoted by the electrolyte solution KHCO_3_. As shown in Scheme 1, for the Fe_2_O_3_-Ti photoanode, under the irradiation of visible light, we used only water and photogenerated h^+^ to produce H_2_O_2_ (R6), because the VB of Fe_2_O_3_ (2.05 eV) was more positive than the potential of H_2_O/H_2_O_2_ (1.76 eV). Meanwhile, based on previous studies [13,28,29], the HCO_3_^-^ anion can act as an effective catalyst for H_2_O_2_ production. The HCO_3_^-^ anion was oxidized by h^+^ to HCO_4_^-^ (R7), which further reacted with H_2_O to form H_2_O_2_ and HCO_3_^-^ (R8).

The the conjectural reaction equations were as follows:

The photocathode:

WO_3_@Co_2_SnO_4_ + hv → h^+^ + e^-^ (1)

O_2_ + e^-^ → ∙O_2_^-^ (2)

O_2_+ 2H^+^ + 2e^-^ → H_2_O_2_ (3)

O_2_^-^ + 2H^+^ + e^-^ → H_2_O_2_ (4)

The photoanode:

Fe_2_O_3_ + hv → h^+^ + e^-^ (5)

2H_2_O + 2h^+^ → H_2_O_2_ + 2H^+^ (6)

or

HCO_3_^-^ + H_2_O → HCO_4_^-^ + 2H^+^ + 2e^-^ (7)

HCO_4_^-^ + H_2_O → H_2_O_2_ + HCO_3_^-^ (8)

## 3. Materials and Methods

### 3.1. Preparation of WO_3_@Co_2_SnO_4_ as Photocathode

The hydrothermal-calcination method was used for the preparation of the WO_3_@Co_2_SnO_4_ heterojunction, and a detailed description of the preparation process of WO_3_@Co_2_SnO_4_ was given in a previous paper [30]. Firstly, NaWO_4_·2H_2_O was dissolved in deionized water (DI) with 4 mL of HCl under stirring, and then, oxalic acid was introduced into the mixture to solubilize a yellow flocculent precipitate. After a hydrothermal reaction at 180 °C for 8 h, the as-prepared products were calcined under 500 °C for 2 h (2 °C·min^−1^) to obtain WO_3_ nanobricks. Secondly, a mixture of CoCl_2_·6H_2_O and SnCl_4_·5H_2_O, with a molar ratio of 2:1, was dissolved in DI, and NaOH (1 mol·min^−1^) and WO_3_ nanobricks were introduced under continuous stirring. Then, the dispersion was transferred to a Teflon-lined steel autoclave for a reaction at 180 °C for 10 h. After calcination at 500 °C for 2 h (2 °C·min^−1^), the WO_3_@Co_2_SnO_4_ was formed.

### 3.2. Preparation of Photoanode Substrate

The activation treatment for Ti-mesh was carried out according to the method described in [31]. Ti-mesh with dimensions of 2 × 3 cm^2^ was impregnated with 0.1 mol·L^−1^ of oxalic acid (C_2_H_2_O_4_) solution at 80 °C for 30 min to remove the surface oxide. Then, the Ti-mesh was dried after rinsing with deionized water.

### 3.3. Preparation of Photocathode

WO_3_@Co_2_SnO_4_ with a mass of 3 mg was dissolved in 0.5 mL deionized water ultrasonically and then dropped on activated carbon paper (2 × 3 cm^2^) [32] until it covered over two-thirds of the paper’s area. After it dried, WO_3_@Co_2_SnO_4_ films to be used as as the photocathode were obtained.

### 3.4. Synthesis of Fe_2_O_3_-Ti as Photoanode

**Scheme 2:**
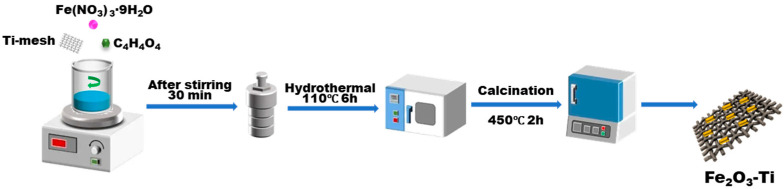
The preparation process of Fe_2_O_3_-Ti for use as the photoanode.

The pretreated Ti-mesh was added into 50 mL of deionized water that contained Fe (NO_3_)_3_·9H_2_O (0.1 mol·L^−1^) and 0.007mol·L^−1^ of fumaric acid (C_4_H_4_O_4_). We performed stirring for 30 min under ambient temperature; then, the mixture was poured into a Teflon-lined steel autoclave and heated constantly at 110 °C for 6h. After the reaction, the autoclave underwent natural cooling, and after centrifugation, the pre-product was thoroughly rinsed with alcohol three times and dried overnight at 70 °C, and then was calcined at 450 °C for 2 h (the heating rate was 2 °C·min^−1^). Finally, Fe_2_O_3_-Ti was obtained to be used as the photoanode material, as exhibited in Scheme 2.

### 3.5. Characterization

The experimental instruments and equipment included a scanning electron microscope (SEM, HITACHI SU-8000, Japan), an X-ray diffractometer (XRD, Bruck D8 Siemens D5000, Germany), a Raman spectrometer (Renishaw, Wotton-under-Edge, UK), a UV-Vis diffuse reflectance spectrometer (DRS, Metash UV-8000S, BaSO_4_ as the reflectance material), and a CHI760E electrochemical station (Shanghai Chenhua, China). X-ray photoelectron spectroscopy (XPS) was conducted through PHI5700 (USA).

### 3.6. Photocatalytic Production of H_2_O_2_ with Photoanode

The production of H_2_O_2_ was performed in a quartz reactor under 300 W Xe light (λ ≥ 420 nm) illumination under a 2V (vs. Ag/AgCl) bias voltage with a three-electrode system, that is, Fe_2_O_3_-Ti as the photoanode, Pt as the cathode, and a Ag/AgCl electrode as the reference electrode. The reaction was carried out with a condensing unit in 1 mol·L^−1^ of KHCO_3_ (20 mL) and 20 °C. Before switching on the power, O_2_ was pumped into the electrolyte under dark conditions with stirring for 30 min to ensure adsorption–desorption equilibrium.

### 3.7. Bidirectional Catalytic Production of H_2_O_2_ by Coupling System

The bidirectional catalytic production of H2O2, whose coupling system used Fe_2_O_3_-Ti and WO_3_@Co_2_SnO_4_ films as the photoanode and photocathode and a Ag/AgCl electrode as the reference electrode, was conducted in a H-type quartz reactor with 300 W Xe light (λ ≥ 420 nm) as the visible-light source. A bias voltage of −0.7 V (vs. Ag/AgCl) was set through a CHI760E electrochemical workstation. A total of 20 mL of KHCO_3_ (1 mol·L^−1^) and 20 mL of Na_2_SO_4_ (0.1 mol·L^−1^) were used as the electrolyte solution for the anode and cathode, respectively. The pH value of the electrolyte solution was adjusted to 3 by using 0.5 mol·L^−1^ HClO_4_. Then, a condensation unit was installed on the coupling system to keep the temperature at 20 °C. Before switching on the power, magnetic stirring with pumping oxygen was performed for 30 min in the dark to achieve absorption–desorption equilibrium. During the reaction process, the concentration of H_2_O_2_ was determined using the potassium iodide method for the anode and the potassium titanium oxalate method for the cathode.

During the reaction process, the concentration of H_2_O_2_ was determined using the titration method; the potassium iodide method was used for the anode and the potassium titanium oxalate method for the cathode.

## 4. Conclusions

In summary, Fe_2_O_3_ with a short rod-like morphology was loaded on Ti-mesh by using the hydrothermal-calcination technique. Fe_2_O_3_-Ti, when used as the photoanode, achieved water oxidation of 110.27 μmol·L^−1^ in two hours for the generation H_2_O_2_ under optimized conditions, including the bias voltage, type of the electrolyte solution, and concentration of the electrolyte. Then, the synergistic photocatalytic production of H_2_O_2_ was investigated using a bidirectional anode–cathode coupling system. A H-type electrolytic tank was established, with a WO_3_@Co_2_SnO_4_ photocathode and an Fe_2_O_3_-Ti photoanode, to produce H_2_O_2_ through the bidirectional photoelectrocatalysis method. The synergistic yield of H_2_O_2_ over a −0.7 V potential reached 919.56 μmol·L^−1^·h^−1^. The PEC reaction mechanism of the bidirectional production of H_2_O_2_ using the double coupling system was discussed and inferred. This work provides an innovative approach for the bidirectional production of H_2_O_2_.

## Data Availability

The data presented in this study are available on request from the corresponding author. The data are not publicly available due to a confidentiality agreement signed at the beginning of the study.
